# “Short Course” of Nonpegylated Liposomal Doxorubicin Plus Paclitaxel and Trastuzumb as Primary Systemic Therapy for Operable and Locally-Advanced Breast Cancer: A Phase II Study (PacLiDox 07)

**DOI:** 10.4021/wjon393w

**Published:** 2011-10-28

**Authors:** D. Rossi, B. Pistilli, D. Morale, Baldelli, V. Casadei, G. Benedetti, P. Alessandroni, V. Catalano, P. Giordani, F. Graziano, S. Luzi Fedeli, G. Fiorentini

**Affiliations:** a Oncology Unit, Marche Nord Hospital, S. Salvatore, Italy; b Oncolgy Unit, Macerata General Hospital, Italy; c Oncology Unit, Ascoli Piceno General Hospital, Italy

**Keywords:** Breast cancer, Neoadjuvant, Liposomal doxorubicin, Paclitaxel, Trastuzumab

## Abstract

**Background:**

Schedules with anthracyclines and taxanes are one of the best options for primary chemotherapy. The addition of trastuzumab showed an impressive percentage of pathological complete responses in Buzdar trial (66.7%). Recently, nonpegylated liposome-encapsulated doxorubicin (NLD) has been widely used in advanced breast cancer with high response rates (98.1 % in Cortes study). The aims of our study were to assess pathological responses and toxicity of NLD plus paclitaxel (and trastuzumab in patients with HER2 overexpression).

**Methods:**

Thirty patients entered the study: 9 locally advanced and 21 operable. Median age was 58.5 years (range: 31-73). 23 patients without HER2 overexpression (or FISH not amplified) were treated with NLD 50 mg/m^2^ every three weeks for 3 courses and weekly paclitaxel 80 mg/m^2^ for 8 courses. 7 patients with HER2 overexpression or FISH amplified were treated with the same schedules plus trastuzumab (Herceptin) 4 mg/kg for the first administration and 2 mg/kg for the following 7 weekly administrations.

**Results:**

Pathological complete response (pCR) was documented in 1 patient (treated with trastuzumab); no residual tumor (infiltrating or “in situ”) on breast was documented in other 2 patients. Objective clinical responses were documented in 22 patients (73.3%): 8 complete, 10 partial and 4 “minimal” responses. 7 patients have shown stable and 1 progressive disease. Clinical response in patients with HER2 overexpression treated with trastuzumab was 100% (4 complete and 3 partial responses). Conservative surgery was performed in 8 (38%) and mastectomy in 13 (62%) out of 21 operable patients; however, 7 out of 14 responding patients with operable disease underwent quadrantectomy (50%). Main toxicity was neutropenia: febrile in 2 patients (7%) and gr. 3-4 in 13 (43%). Other grade 3 toxicities were as follows: vomiting in 1 patient, asthenia in 1 patient, joint symptom in 1 patient. 3 patients were withdrawn from the study. No episodes of left ventricular ejection fraction (LVEF) < 50% were recorded (with a median reduction of 8%).

**Conclusions:**

A “short course” of paclitaxel and NLD is active in terms of clinical response and conservative surgery for patients with potentially operable and locally advanced breast cancer; toxicity was manageable. High activity of the combination with trastuzumab has been confirmed. However, with this “short course” schedule, the result in term of clinical responses didn't turn into complete pathological responses.

## Introduction

Breast cancer is the most common form of malignancy in women in the Western world. In the European Union in 1990, there were approximately 179 000 new cases (28% of all cancers in females) and 74 000 recorded deaths (15% of cancer deaths) [1]. Surgery with or without radiotherapy can control local-regional disease in the majority of patients. However, more than 60% will ultimately die due to distant disease recurrence. Neoadjuvant chemotherapy in conjunction with radiotherapy and surgery is the treatment of choice for patients with locally advanced, inoperable breast cancer and recently this approach has been extended to patients with large operable tumors [2]. In operable breast cancer both randomized and non-randomized trials generally reported high response rates (> 50% and often 90% or higher) to neoadjuvant chemotherapy and an increase in breast conserving surgery. Improvements in survival and disease-free survival have generally not been shown, although no disadvantage has been shown for neoadjuvant therapy, either. Conventional doxorubicin played an important role in the treatment of breast cancer patients and remains a mainstay of therapy in adjuvant and neoadjuvant settings [3, 4]. The combination of doxorubicin and paclitaxel is widely used in advanced disease [5] and demonstrated its efficacy when shifted toward preoperative chemotherapy [6]. Unfortunately, the development of a chronic cumulative dose-dependent cardiomyopathy limits the use of anthracyclines; the risk of congestive heart failure increasing sharply with lifetime cumulative doses over 450 mg/m^2^. Liposomal encapsulation is a method to reduce this peculiar toxicity preserving antitumor activity. NLD documented less cardiac toxicity and mucositis than conventional doxorubicin without hand-foot syndrome: two studies have shown comparable overall survival in metastatic breast cancer with reduced cardiac toxicity [7, 8]. In these last years, a monoclonal antibody (trastuzumab) has become a milestone in metastatic breast cancer and, recently, in preoperative setting. In Buzdar trial [9], with 4 cycles of paclitaxel followed by 4 cycles of FEC and trastuzumab, was documented an impressive 66.7% of pCR. In NOAH trial [10], trastuzumab was combined with a sequential treatment of Adriamycin/paclitaxel (3 cycles), paclitaxel (4 cycles), CMF (3 cycles) and compared with the same schedules without trastuzumab: pCR was 38% versus 19% and 3-year event-free survival was 71% versus 56%. In a study by Cortes et al [11], NLD was combined with weekly paclitaxel and trastuzumab in 54 locally advanced/metastatic breast cancer patients with 98.1% of overall response rates; no patients developed symptomatic cardiac heart failure (a decline in LVEF was recorded in 12 patients); the main toxicities were mucositis (57.4%), nausea (50%), asthenia (51.8%) and neutropenia (29.6%). This study confirmed the possibility to combine NLD and trastuzumab with safety in term of cardiac toxicity. However, to the best of our knowledge, no studies have been published in preoperative setting with a schedule including NLD, paclitaxel with or without trastuzumab. We have published in 2008 another study [12] with pegylated liposomal doxorubicin and weekly paclitaxel demonstrating good activity in terms of clinical response rates (74%) and conservative surgery (55%); the pathological responses were not impressive (9%). The main toxicity was hand-foot syndrome in 13 patients (37%). Mantenaing the same “short course”of paclitaxel (80 mg/m^2^/weekly for 8 cycles), in 2007 we thought to plan a phase II study replacing pegylated with nonpegylated liposomal doxorubicin and combining these drugs with trastuzumab in order to improve pCR (which was the first aim of the trial) and the safety profile. Other aims of the study were clinical response rates and percentage of conservative surgery.

## Patients and Methods

### Eligibility criteria

Thirty patients with histologically confirmed breast cancer entered the study. Patients with tumor size, mammographically detected, larger than 2 cm, with or without axillary palpable lymph nodes were enrolled. The study included patients with operable disease but not candidates, particularly for breast size, to conservative surgery at the time of diagnosis (T2 N0; T2-3 N0-1; T2-3 N1-2) and patients with locally advanced breast cancer (T4a, b, c, N0-3; T4d); a fine needle aspiration was not performed in patients with palpable axillary lymph nodes. After mammography and core needle biopsy, all patients underwent disease staging with a complete blood count, chemistry profile, chest x-ray, liver ultrasound and bone scan. HER2 status was detected with immunochemistry method (DAKO-test); in patients with DAKO-test 2+, FISH amplification was performed. Other selection criteria included: (a) age 18 years or elder (less than 75 years), (b) ECOG performance status 0-1, (c) adequate renal, liver and bone marrow function, (d) a life expectancy of at least 12 months. Exclusion criteria included: (a) coexistent diagnosis of ischemic cardiopathy or other cardiopathy (an evaluation of left ventricular ejection with ultrasound or MUGA scan was performed before neoadjuvant and after the completion of chemotherapy), (b) previous treatment for breast cancer, including surgery, radiation, cytotoxic and endocrine treatment, (c) previous cancer except for curatively treated non-melanoma skin cancer or carcinoma “in situ” of the cervix, (d) peripheral neuropathy, (e) active infection or other serious medical or psychiatric condition which would impair the ability of patient to receive protocol treatment. The primary objective was the pathological complete response of a “short course” of NLD with weekly paclitaxel (plus trastuzumab in patients with HER2 overexpression) in locally advanced and operable breast cancer; secondary objectives were the evaluation of clinical responses, toxicity and the rate of conservative surgery. A written informed consent was obtained from all patients. Local Ethical Committee approved the study (EudraCT number: 2008-001369-27).

### Study design

The schedule consisted of NLD 50 mg/m^2^ every three weeks for 3 courses and weekly paclitaxel 80 mg/m^2^ for 8 courses. Patients with HER2 overexpression or FISH amplified were treated with the same schedules plus trastuzumab 4 mg/kg for the first administration and 2 mg/kg for the following 7 weekly administrations. Pathological response has been assessed with standard histological procedures. Objective response was evaluated with clinical examination before neoadjuvant and after the completion of chemotherapy according to WHO criteria [13]. Patients with complete or partial response of breast tumor were evaluated for conservative surgery, except for patients with T4a, b, c, d (thus, the percentage of breast-conserving surgery has been evaluated only in operable patients). Regardless clinical response, the surgeon of each oncology center could independently decide for quadrantectomy or mastectomy according their expertise. Responding patients were treated with adjuvant chemotherapy consisted of doxorubicin/cyclophosphamide, doxorubicin/cyclophosphamide followed by paclitaxel, epirubicin and paclitaxel (concomitant or sequential); a docetaxel-based chemotherapy was administered in the other patients. Patients with HER2 overexpression were treated with adjuvant trastuzumab every three weeks (6 mg/kg) to complete 1 year of treatment. The schedules administered were: AC (doxorubicin 60 mg/m^2^ and cyclophosphamide 600 mg/m^2^) for 4 cycles; AC for 4 cycles followed by paclitaxel (175 mg/m^2^) for 4 cycles; epirubicin (90 mg/m^2^) plus paclitaxel (175 mg/m^2^) for 4-6 cycles; Epirubicin (100 mg/m^2^) for 4 cycles followed by paclitaxel (175 mg/m^2^) for 4 cycles; docetaxel (100 mg/m^2^) for 6 cycles; TAC (docetaxel 75 mg/m^2^, doxorubicin 50 mg/m^2^; cyclophosphamide 500 mg/m^2^) for 6 cycles. After adjuvant chemotherapy, premenopausal patients with hormone-receptor positive, started hormonal therapy with tamoxifen (20 mg/day for 5 years) and LH-RH analogue (for 2 years); postmenopausal patients started an aromatase inhibitor for 5 years. Patients with a conservative surgery and patients with a baseline T4-tumor were considered for radiotherapy on residual breast or chest wall, respectively. Surgical specimens were evaluated for pathological response on breast and axillary lymph-nodes. The disappearance of infiltrating or “in situ” tumor cells on breast and axilla was considered as pCR.

### Dose reduction

A blood count and chemistry profile were performed the day before chemotherapy. In the case of grade 3 and 4 neutropenia or thrombocytopenia, treatment was delayed until the absolute neutrophil count was ≥ 1500/mm^3^ and platelets count was ≥ 100,000/ mm^3^ (for patients with grade 4 neutropenia, G-CSF administration was permitted for at least 3 days or until complete neutrophil recovery, at the dose of 30 micrograms every day subcutaneously). In the case of grade 4 hematological toxicity, the dose of nonpegylated liposomal doxorubicin and paclitaxel was reduced by 25% in the subsequent cycles. The dose of the two drugs could be reduced by 25% for any grade 3 non-hematological toxicity; treatment was permanently interrupted for any grade 4 non-hematological toxicity. Toxicity profile was evaluated according to NCI-CTC criteria v3.0.

### Statistical Design

The aim of this phase II study is to determine the pathological complete response rate on breast and axilla. Simon’s method has been used to calculate the sample size. Considering the optimal two stage design for phase II studies, with the difference p1-p0 = 15% between “standard” chemotherapy (p0 = 10%) and the “new” therapy (p1 = 25%), a fixing error probabilities (α = 0.05 and β = 0.20), the number of patients for the first step was 18. If ≤ 2 complete pathological responses were seen in these first 18 patients, then the trial was stopped. Otherwise, the accrual would be continued to a total of 43 patients. The average sample size is 24.7 and probability of early termination is 0.73 for therapy with a response probability of 0.05.

## Results

Thirty patients entered the study; all were evaluable for clinical, pathological response and toxicity. 9 locally advanced (IIIB) and 21 operable (6 IIA, 4 IIB, 11 IIIA). Median age was 58.5 years (range: 31-73), 12 premenopausal and 18 postmenopausal patients. Biological features were: EgR positive in 25 patients with median of 90% (range 30-100%), EgR and PgR negative in 4 patients (Table 1). Median Ki-67 was 30% (range 5-90%). 23 patients without HER2 overexpression (or FISH not amplified) were treated with NLD 50 mg/m^2^ every three weeks for 3 courses and weekly paclitaxel 80 mg/m^2^ for 8 courses. 7 patients with HER2 overexpression or FISH amplified were treated with the same schedules plus trastuzumab 4 mg/kg for the first administration and 2 mg/kg for the following 7 weekly administrations. pCR was documented in 1 patient (with HER2 overexpression and treated with trastuzumab); no residual tumor (infiltrating or “in situ”) on breast was documented in other 2 patients. Objective clinical responses were documented in 22 patients (73.3%): 8 complete, 10 partial and 4 “minimal” responses. 7 patients showed stable and 1 progressive disease (Table 2). Clinical response in 7 patients with HER2 overexpression treated with trastuzumab was 100% (4 complete and 3 partial responses). Conservative surgery was performed in 8 (38%) and mastectomy in 13 (62%) out of 21 operable patients. 7 out of 14 responding patients with operable disease underwent quadrantectomy (50%). Main toxicity was neutropenia: febrile in 2 patients (7%) and gr. 3-4 in 13 (43%). No episodes of LVEF < 50% was recorded (median reduction of LVEF was 8%). Other grade 3 toxicities were as follows: vomiting in 1, asthenia in 1, joint symptoms in 1 patient (Table 3). Growth-Colony Stimulating Factors were delivered in 9 patients (30%) and epoietin in 1 patient; a dosage reduction was necessary in 8 patients (26.6%) due to hematological toxicity. 3 patients (10%) were withdrawn from the study treatment: 1 due to progressive disease, 1 due to fever following venous catheter infection, 1 due to pulmonary embolism following a deep venous thrombosis.

**Table 1:** Characteristics of Patients

Patients	Numbers/value of characteristics
Total	30
Age (median)	58.5 (31-73)
Baseline stage	
IIA	6
IIB	4
IIIA	11
IIIB	9
Menopausal Status	
Post	18
Pre	12
Hormonal Receptor	
EgR positive	25
EgR negative	5
EgR/PgR negative	4
HER2-status	
0	9
1	9
2	5*
3	7

^*^Fluorescence In-Situ Hybridation not amplified.

**Table 2:** Response

	Number of patients	Percentage (%)
Complete response	8	27
Partial response	10	33
Minimal response	4	13
Stable disease	7	24
Progressive disease	1	3
pCR	1	3
No residual tumor on breast	2	6

**Table 3:** Toxicity

	Gr. 2	Gr. 3	Gr. 4
Neutropenia	5	10*	3*
Anemia	1	0	0
Nausea	8	0	0
Vomiting	0	1	0
Joint symptoms	3	1	0
Asthenia	0	1	0
Diarrhoea	1	0	0
Neuropathy	2	0	0
Hepatotoxicity	4	0	0

^*^1 febrile neutropenia.

## Discussion

The combination of anthracyclines and taxanes are the better choice when a preoperative chemotherapy has planned. Activity of this schedule has been documented in a number of phase II and III studies with complete clinical responses varied from 9% to 34% and pathological complete responses ranged from 5% to 27.5% in all patients [14]. For example, 536 patients with large (> 2.5 cm) primary tumors were treated in two prospective, non-randomized trials at the Milan Cancer Institute. 85% of patients were able to undergo breast-sparing surgery and in 3% of patients, surgical specimens showed no residual tumor [15]. In the largest trial to date (NSABP B-18), 1523 women with stage I-III breast cancer were randomized to receive preoperative or postoperative chemotherapy, consisting of 4 cycles of doxorubicin and cyclophosphamide. Tumor size reduced in 80% of patients receiving preoperative chemotherapy and 36% had a clinical complete response (CR) [16]. Overall, primary chemotherapy was able to decrease the incidence of pathologically negative nodes (59% versus 43%); however, no difference in disease-free survival, distant disease-free survival or overall survival was documented [17]. Moreover, patients whose tumors overexpress HER2 may derive particular benefit from anthracyclines. In a study of 638 patients with node-positive, hormone receptor-negative breast cancer enrolled in the National Surgical Adjuvant Breast Project (NSABP) B-11 trial, patients with HER2 overexpression had a statistically superior disease-free survival if treated with a doxorubicin-containing regimen compared with a similar regimen without doxorubicin [18]. Another retrospective study, which examined patients enrolled in NSABP B-15 trial (in which patients were randomized to AC alone, versus AC followed by CMF, versus CMF alone) found HER2 overexpression in 599 (29%) of 2034 stained tumor sections. Among HER2 positive women, those treated with AC had a trend toward improved DFS compared with those who received only CMF [19]. However, these combinations have a low toxicity profile with a number of side effects such as hematological, cardiological, neurological and gastrointestinal (mucositis) toxicity. Cardiac toxicity due to conventional anthracylines is a worrying side effect because, unlike the other adverse events, is not a temporary damage but could determine an irreversible injury of myocardium. The dose-dependent nature of doxorubicin-induced cardiac toxicity has been well described with overt congestive heart failure increasing from less than 10% at a cumulative doxorubicin dose of 500 mg/m^2^, to 15% at 600 mg/m^2^ and 30-40% at 700 mg/m^2^ [20, 21]. One of the hypotheses suggesting mechanisms for this myocardial damage is the high peak plasma level of drug accompanying a standard 15-30 minute infusion of doxorubicin every three weeks [22]. This is supported by evidence that dosing schedules designed to lower peak plasma concentration reduce cardiotoxicity. Liposomal encapsulation of anthracyclines is one method of lowering peak plasma concentration, and it has been shown in a number of animal models that this reduces cardiotoxicity [23]. NLD is a form that was designed to increase the amount of drug delivered to tumors and decrease the amount going to healthy organs, such as the heart. In the TLC D-99 model, doxorubicin is pulled into the interior of the vesicle by the generation of an electropotential across the liposome membrane. This mechanism for remote loading involves the generation of a pH gradient between the inside of the liposome and the extra-liposomal buffer. Harris et al performed a randomized multicenter trial that compared NLD with conventional doxorubicin at the same doses (75 mg/m^2^) as first-line therapy in metastatic breast cancer. Protocol-defined cardiotoxicity was observed in 13% of NLD compared to 29% of doxorubicin patients (with 2 versus 9 cases of congestive heart failure). Median cumulative doxorubicin dose at onset of cardiotoxicity was 785 mg/m^2^ for NLD versus 570 mg/m^2^ for doxorubicin. Myelosuppression was the most frequent and severe toxicity and it was comparable in both treatment groups, but infections (grade 3) were less frequent in NLD group. Antitumor activity was comparable [7]. In another phase III study metastatic breast cancer patients were randomized to receive NLD 60 mg/m^2^ and cyclophosphamide 600 mg/m^2^ (MC) or doxorubicin 60 mg/m^2^ and cyclophosphamide 600 mg/m^2^ (AC). NLD improved the therapeutic index of doxorubicin by significantly reducing cardiotoxicity (6% MC versus 21% AC) and grade 4 neutropenia (61% MC versus 75% AC). Response rates were 43% in both treatment groups [8]. Recently, a monoclonal antibody (trastuzumab) has become a “gold standard” in preoperative chemotherapy improving complete pathological response rates [9, 10]. Buzdar and NOAH trials are particularly important for another reason: they confirmed the possibility to combine trastuzumab and anthracyclines with safety (in NOAH trial, only 2 out of 117 patients in trastuzumab arm had a grade 2 decrease of LVEF and other 2 had a reversible grade 3 decrease). In a phase I dose-finding study [24], NLD has been combined with trastuzumab in 40 advanced breast cancer patients: the dose for phase II studies was 60 mg/m^2^ for NLD and trastuzumab 4 mg/kg (first) and 2 mg/kg (following administrations). Cardiac toxicity was highly manageable: only 5 patients (13%) developed left ventricular ejection fraction reductions < 50% (4 were pretreated with doxorubicin); gr.3-4 neutropenia was the main toxicity (65%). Objective tumor response occurred in 50% of evaluable patients. A regimen of NLD 50 mg/m^2^, paclitaxel 80 mg/m^2^ and trastuzumab 4 mg/kg followed by 2 mg/kg was delivered in 54 previously untreated patients with HER2-positive, locally advanced and metastatic breast cancer [11]. The overall response rate was 98.1% (complete 53.7% and partial 44.4%). 16 out of 28 patients with locally advanced breast cancer underwent surgery (57.1%): two of these obtained a pathological complete response (12.5%). The most common adverse events included alopecia (85.1%), mucositis (57.4%), nausea (50%), and asthenia (58.1%). 11 patients were withdrawn from the study due to adverse events including neutropenia and febrile neutropenia (7.4%), neuropathy (1.8%), bilateral pneumonia and neutropenia (1.8%), dermatitis (3.7%), decrease in performance status (1.8%) and pancreatitis (1.8%). 8 additional patients were withdrawn due to asymptomatic protocol-defined cardiac adverse events.

A similar combination with docetaxel (75 mg/m^2^) instead of paclitaxel [25] has been administered in 31 patients with metastatic breast cancer: overall response rate was 65.5% and the most common adverse events were hematological toxicity, alopecia, asthenia and fever (cardiotoxicity developed in three patients during treatment and in another two after the end of the study). To the best of our knowledge, our study is the only conducted even in patients with operable breast cancer. The results, in term of pCR, was not impressive (only 1, in a patient treated with trastuzumab) and the first aim of the study was not reached. However, in 2 patients no residual tumor has been found on breast histological specimens (1 of these patients had a tumor diameter of about 8 cm). Nevertheless, in terms of clinical response and conservative surgery, the combination was active with tumor down-staging in 73.3% of patients and quadrantectomy in 7 out of 14 responding patients (50%). This latter percentage would be increased to 86% if the other 5 operable patients with complete clinical response had been addressed to conservative approach but the surgeon of one single center independently decided to perform mastectomy, regardless chemotherapy response. The combination with trastuzumab confirmed high activity of the schedule with 100% of response rates (4 complete and 3 partial). Neutropenia was the main toxicity (grade 3-4 neutropenia in 13 patients: 43%) but manageable with G-CSF (9 patients); only two episodes of febrile neutropenia, without hospitalization, were recorded. Moreover, cardiac toxicity was not an issue considering that no patients reported a decrease in LVEF lower than 50% (median reduction of 8%). Concerning the low rate of pCR, we emphasize two issues: the duration of treatment and the EgR expression. Now we think that a course of two months is too short to reach a suitable percentage of pCR and the only replace of pegylated with nonpegylated liposomal doxorubicin isn't enough, even though allow to avoid hand-foot syndrome (in our previous study, we obtained a pCR only in 3 out of 35 patients). The number (25/30) and the high EgR expression of our patients (median 90%) could have reduced chemotherapy response. A number of studies [26, 27, 28] demonstrated that the incidence of pCR were associated with absence of estrogen receptor expression. In Colleoni study [27], patients with EgR/PgR-absent tumors were 12.0 times more likely to achieve a pathological complete response (P < 0.0001). A comparison between our study and other trials is hard because we did not find similar published trials. Analyzing the study by Cortes [11] and Theodoulou [24], we can draw that the combination of NLD with trastuzumab is feasible, safety in term of cardiac toxicity and active in term of response rates. When paclitaxel added, toxicity increased especially for myelosuppression.

In summary, we think that our study showed that the combination of NLD with weekly paclitaxel was active in terms of clinical response and conservative surgery confirming high activity of trastuzumab in preoperative settings (although only in 7 patients). This “short course” of treatment reduced overall toxicity but was not enough to achieve a suitable pathological complete response rates.
